# P-1141. Antibiotic administration for *Staphylococcus aureus* infections in US children's hospitals, 2015-2023

**DOI:** 10.1093/ofid/ofae631.1328

**Published:** 2025-01-29

**Authors:** Tyler Walsh, Fnu Shambhavi, Jason G Newland, Stephanie A Fritz

**Affiliations:** Washington University School of Medicine in Saint Louis, St. Louis, Missouri; Washington university in St Louis, St Louis, Missouri; Washington University in St. Louis School of Medicine, St. Louis, Missouri; Washington University School of Medicine, Saint Louis, MO

## Abstract

**Background:**

The epidemiology of methicillin-resistant *Staphylococcus aureus* (MRSA) and methicillin-susceptible *S. aureus* (MSSA) infections in children has changed. In response to the introduction of new antibiotics published clinical data, treatment strategies have evolved. The objective of this study is to describe the antimicrobial treatment regimens for children hospitalized with this important pathogen.

**Table 1.**
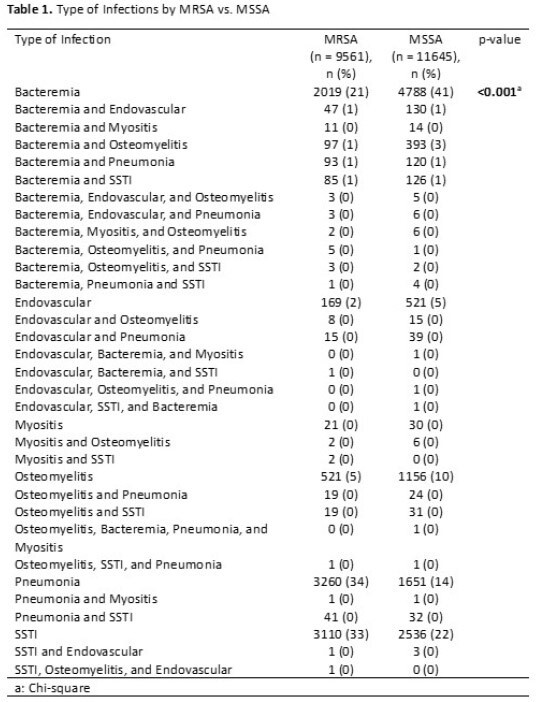
Type of Infections by MRSA vs. MSSA

**Methods:**

A retrospective observational study was performed using the Pediatric Health Information System (PHIS) data, including all patients discharged from a PHIS-member hospital from October 1, 2015 to June 31, 2023. Patients with ICD 10 codes identifying an MRSA or MSSA infection and indicating the type of infection were included (Table 1): bacteremia, endovascular, pneumonia, skin and soft tissue infection (SSTI), osteomyelitis, and myositis. The proportion of anti-staphylococcal antibiotics of interest received by all patients, patients with MRSA infections, and patients with MSSA infections were calculated by year.

**Figure 1.**
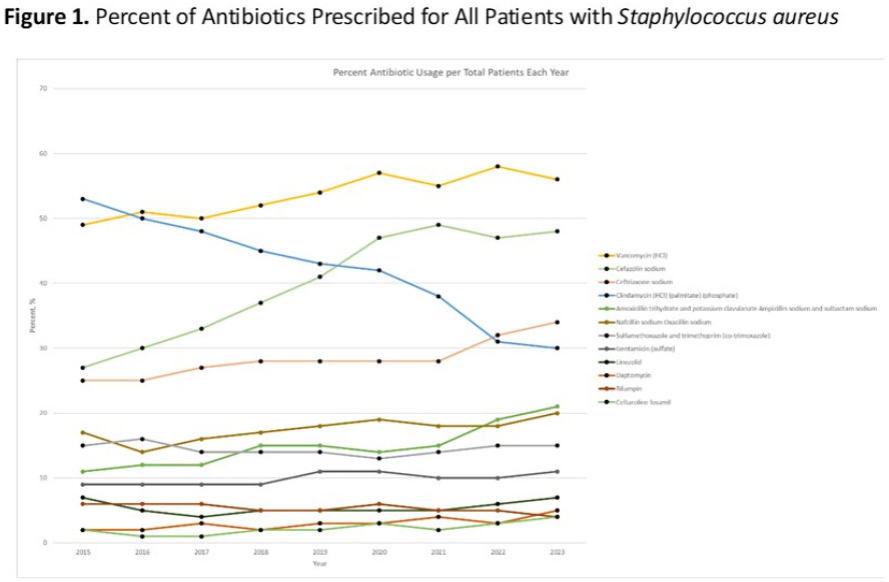
Percent of Antibiotics Prescribed for All Patients with Staphylococcus aureus

**Results:**

21,206 patients met eligibility criteria and were included in the final data set. Infections due to MRSA and MSSA were 9,561 and 11,645, respectively (Table 1). The proportion of patients with *S. aureus* infections caused by MRSA decreased from 2015 (55%) to 2023 (41%). The most frequent infections were bacteremia (32%), SSTI (27%), and pneumonia (23%, Table 1). The most common antibiotics prescribed for all *S. aureus* infections in 2015 were clindamycin (53%), vancomycin (49%), and cefazolin (27%) and in 2023 were vancomycin (56%), cefazolin (48%), and ceftriaxone (34%, Figure 1). While vancomycin prescriptions remained consistent over the study period, clindamycin administration decreased 20% (Figure 1). Daptomycin and ceftaroline, the newest MRSA agents, increased from 2% and 3% to 7% and 8%, respectively, of agents prescribed for MRSA infections (Figure 2). For MSSA infections, cefazolin increased in use while oxacillin/nafcillin use remained consistent (Figure 3).

**Figure:**
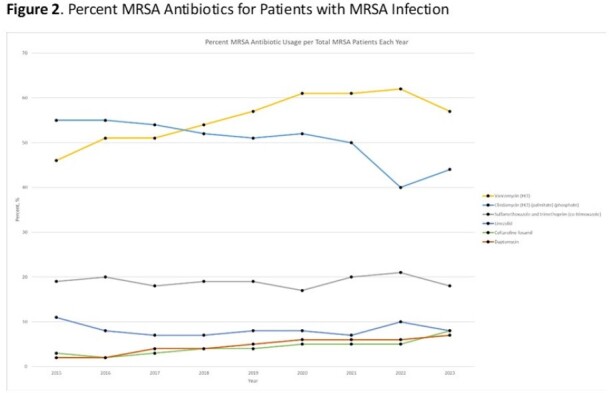

**Conclusion:**

Clindamycin administration has substantially decreased, likely a result of increased resistance prevalence in both MSSA and MRSA. Cefazolin use increased commensurate with the increasing incidence of MSSA infection.

**Figure:**
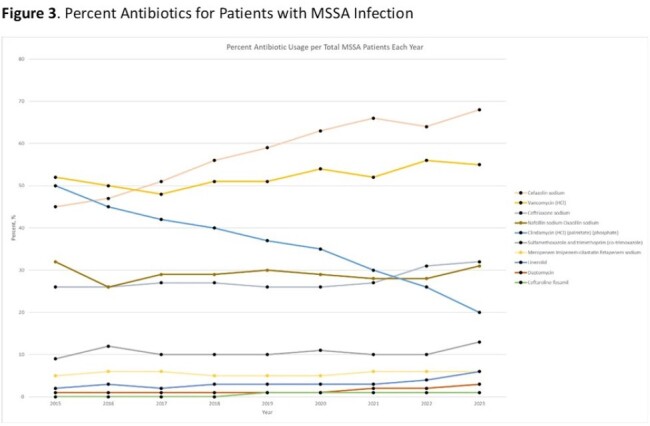

**Disclosures:**

**Jason G. Newland, MD, MEd**, Moderna: Grant/Research Support|Pfizer: Grant/Research Support

